# Crystal structure and Hirshfeld surface analysis of 2-methyl­quinazolin-4(3*H*)-one hydro­chloride

**DOI:** 10.1107/S2056989025000258

**Published:** 2025-01-17

**Authors:** Muzaffar Davlatboev, Sevara Allabergenova, Fazliddin Zulpanov, Ubaydullo Yakubov, Akmaljon Tojiboev, Tulkinjon Sattarov

**Affiliations:** a Namangan State University, Boburshoh str. 161, Namangan, 160107, Uzbekistan; b Institute of the Chemistry of Plant Substances, Uzbekistan Academy of Sciences, Mirzo Ulugbek Str. 77, Tashkent 100170, Uzbekistan; c University of Geological Sciences, Olimlar Str. 64, Tashkent 100170, Uzbekistan; Vienna University of Technology, Austria

**Keywords:** quinazolin-4-one, crystal structure, hydrogen-bonding, inter­mol­ecular inter­actions, organic salt

## Abstract

The title salt (systematic name: 2-methyl-4-oxo-3,4-dihydroquinazolin-1-ium chloride), C_9_H_9_N_2_O^+^·Cl^−^, has ortho­rhom­bic (*Pbcm*) symmetry. Except for two methyl H atoms, all atoms of the mol­ecular cation are located about a mirror plane, making the quinazolinium moiety exactly planar. Individual mol­ecules are arranged in (001) layers in the crystal. Supra­molecular features include N—H⋯Cl hydrogen-bonding inter­actions, leading to zigzag chains along [010] with *D*_1_^1^(2) and *C*_1_^2^(6) graph-set motifs. Additionally, weak π–π stacking inter­actions occur between benzene rings in adjacent layers. Hirshfeld surface analysis revealed that the most important contributions to the surface contacts are from H⋯H (36.1%), H⋯C/C⋯H (25.8%), and H⋯O/O⋯H (17.7%) inter­actions.

## Abstract 2

The quinazolinium moiety of the organic cation is located about a mirror plane. In the crystal, individual cations are linked into [010] zigzag chains by N—H⋯Cl hydrogen-bonding inter­actions.

## 1. Chemical context

Syntheses based on pyrimidines (quinazolines) condensed with a benzene ring are widely used in agricultural and medical practice (Zayed, 2023 ▸). In particular, drugs based on compounds of this class are used against viruses, microbes, colds and cancer (Li *et al.*, 2021 ▸; Arachchige & Yi, 2019 ▸) as well as stimulants and pesticides (Alsibaee *et al.*, 2023 ▸). Examples of such types of drugs that have been used successfully against various types of cancer in recent years are *imatinib*, *erlotinib*, *lapatinib* and *afatinib*. Therefore, targeted syntheses of biologically active compounds containing this pharmacophore (*i.e.* the quinazoline ring), are important to determine their physical, chemical and biological properties. In this context, we report here the mol­ecular and crystal structures of 2-methyl quinazolin-4(3*H*)-one hydro­chloride (**I**) and its Hirshfeld surface analysis.
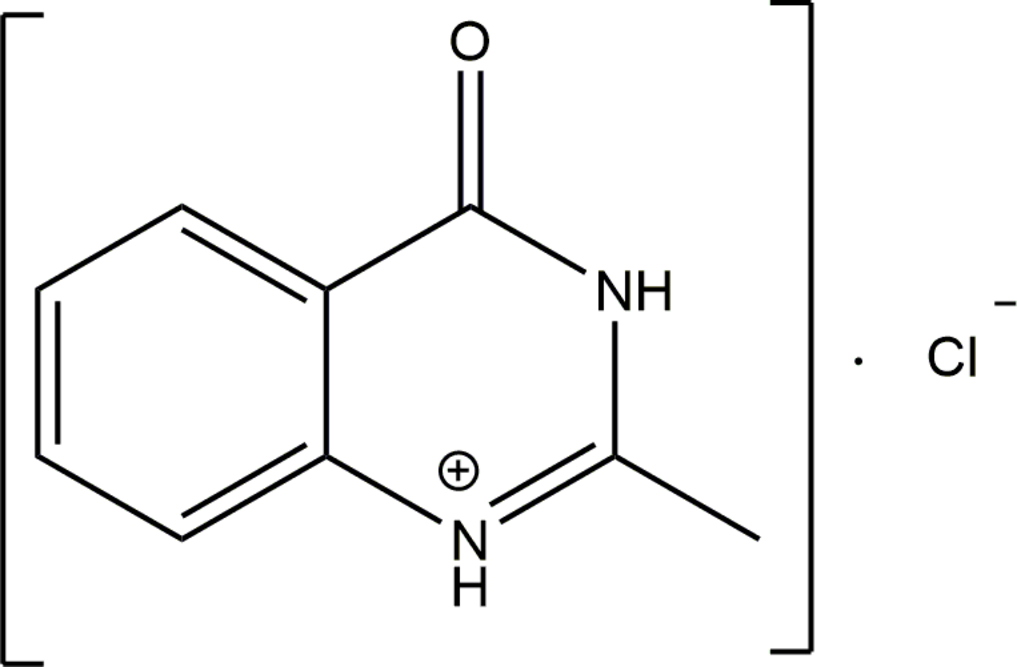


## 2. Structural commentary

**Figure 1:**
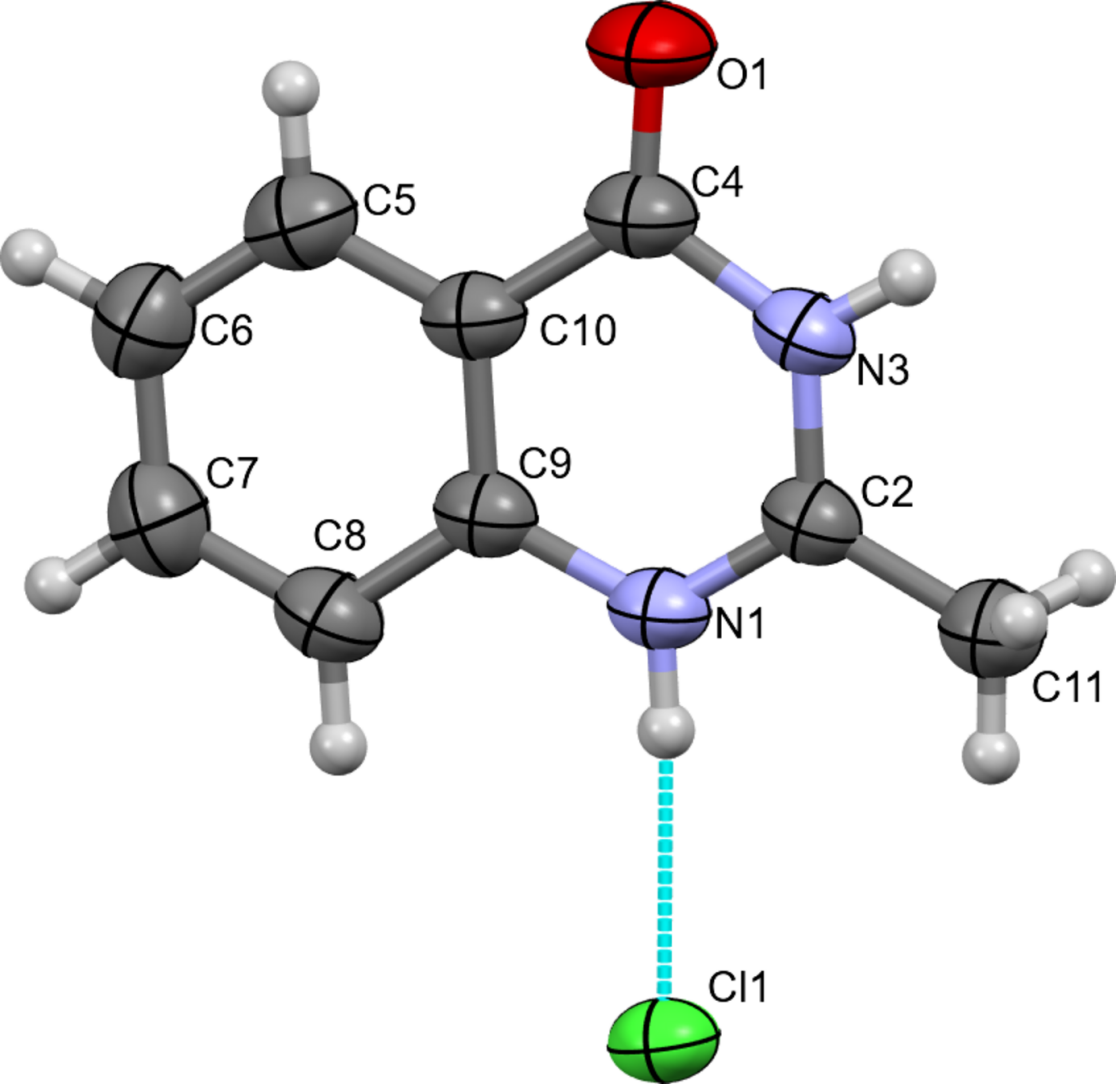
The asymmetric unit of (**I**) with displacement ellipsoids drawn at the 50% probability level. The dotted turquoise line represents an N—H⋯Cl hydrogen bond.

**Figure 2:**
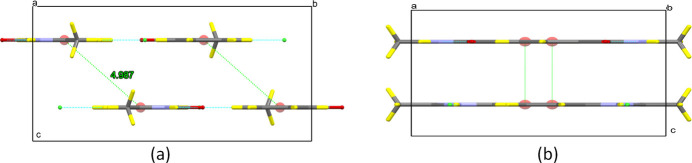
Packing of (**I**) (*a*) along the *a* axis and (*b*) along the *b* axis, showing the π–π inter­actions.

The asymmetric unit of (**I**) consists of a quinazolinium cation and a Cl^−^ anion (Fig. 1 ▸). Except for methyl H atom H11*b* and its symmetry-related counterpart, all atoms are located on a mirror plane, making the benzene and pyrimidine rings in the cation exactly planar (Fig. 2 ▸). The basic heteroatom N1 of the pyrimidine ring is protonated, and the resulting positive charge is delocalized within the –N—C—N– moiety in the ring, making the C2—N1 and C2—N3 bonds shorter than the C4—N3 and C9—N1 bonds. Similar differences were observed in related compounds reported in the literature (Sharma *et al.*, 1993 ▸; Turgunov *et al.*, 2003 ▸; Tozhiboev *et al.*, 2005 ▸, Tojiboev *et al.*, 2021 ▸).

## 3. Supra­molecular features

**Figure 3:**
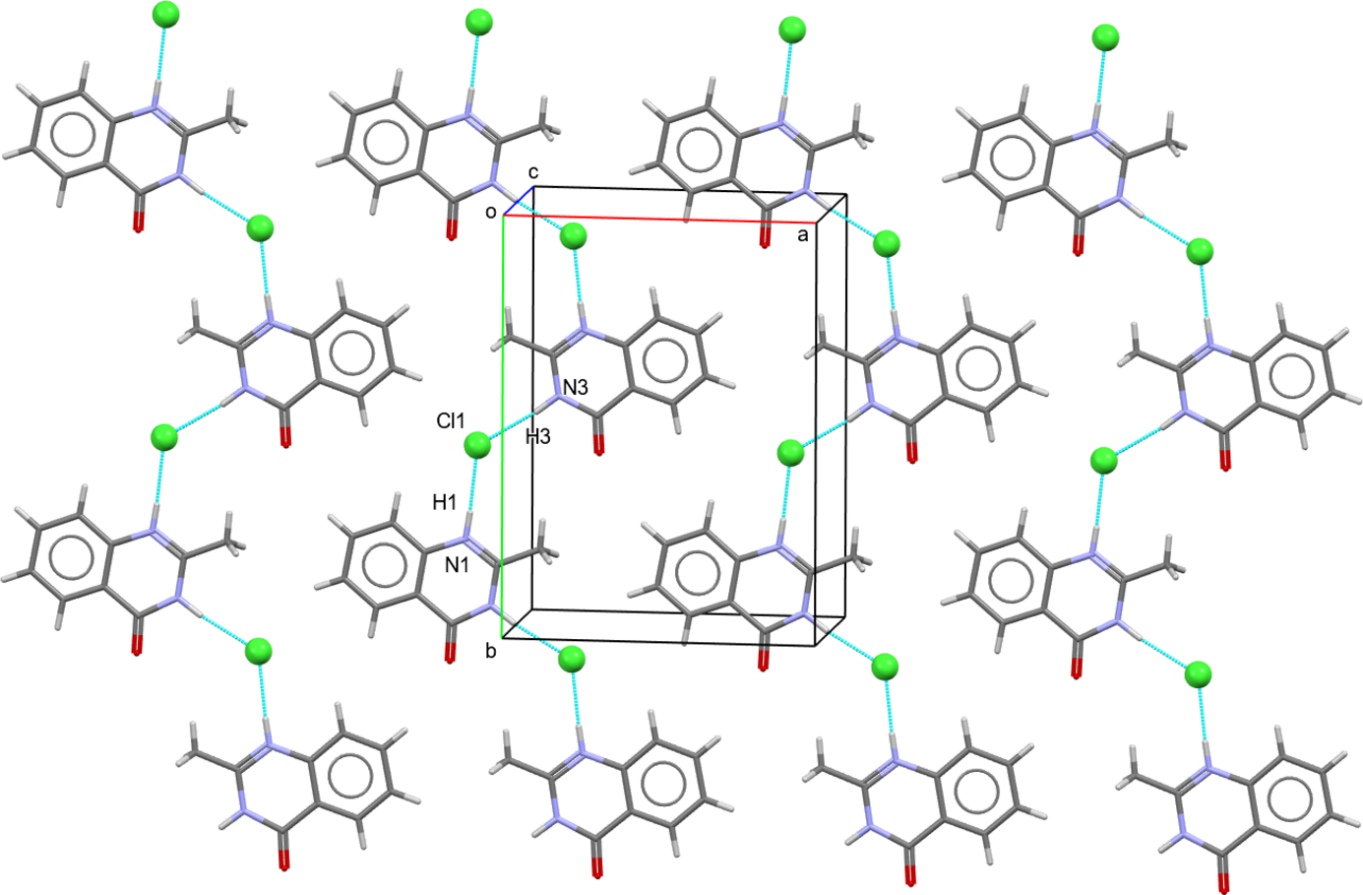
Packing of (**I**) along the *c* axis. Hydrogen bonding between N1—H1⋯Cl and N3—H3⋯Cl is shown as blue dotted lines.

In the crystal of (**I**), the cationic mol­ecules are arranged in flat (001) layers. Individual mol­ecules are linked to Cl^−^ anions through N—H⋯Cl hydrogen-bonding inter­actions (Table 1 ▸) into zigzag chains extending parallel to [010] (Fig. 3 ▸), generating *D*_1_^1^(2) and 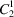
(6) graph-set motifs (Bernstein *et al.*, 1995 ▸). In addition, weak highly slipped π–π stacking inter­actions (Fig. 2 ▸) occur between benzene (centroid *Cg*2) rings in adjacent layers and involve contact distances *Cg*2⋯*Cg*2(1 − *x*, 1 − *y*, 1 − *z*) of 4.987 (14) Å (slippage 3.280 Å).

## 4. Hirshfeld surface analysis

**Figure 4:**
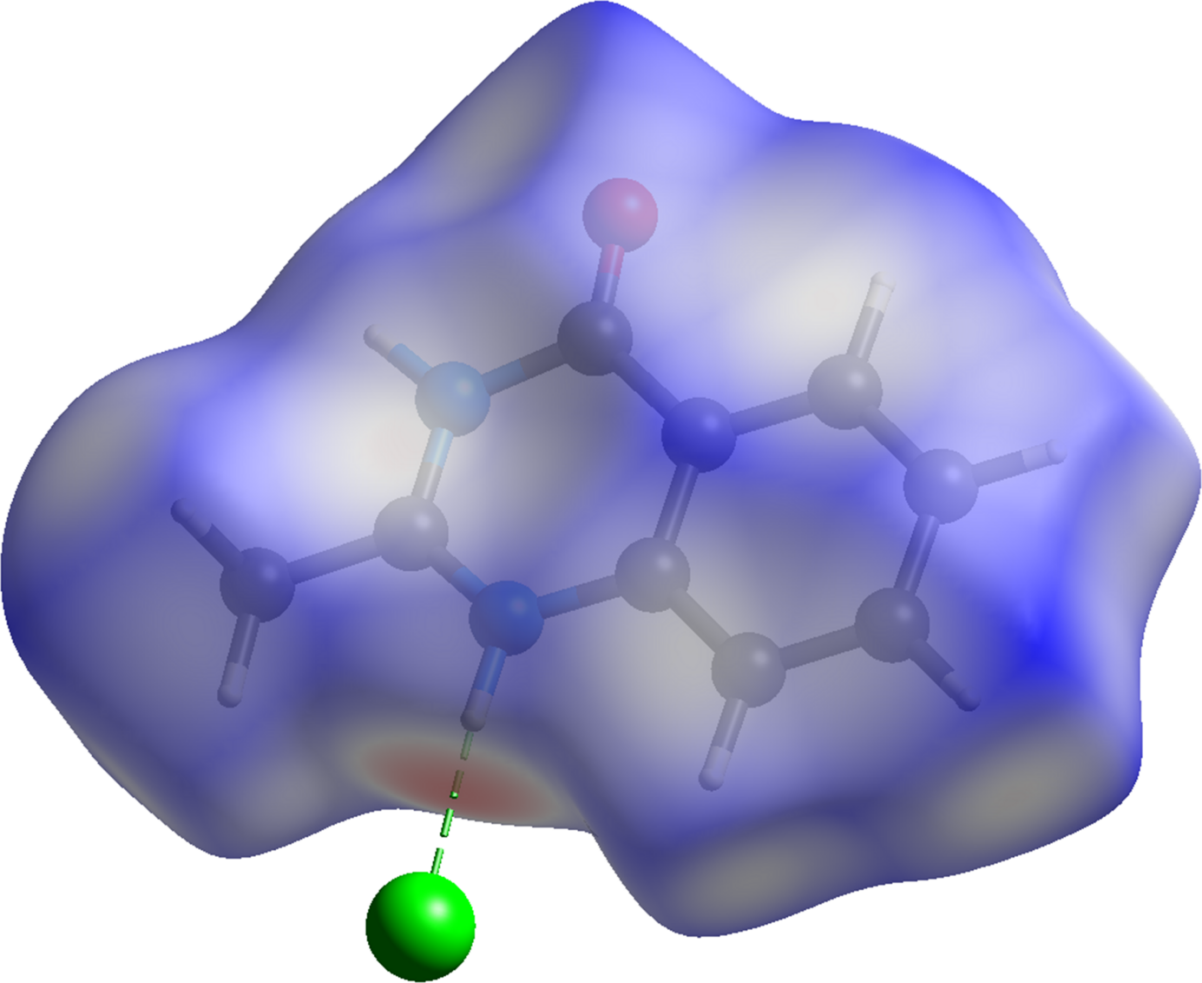
Three-dimensional Hirshfeld surface of (**I**) mapped over *d*_norm_.

A Hirshfeld surface analysis (Hirshfeld, 1977 ▸) was carried out using *CrystalExplorer* (Spackman *et al.*, 2021 ▸) to visualize non-covalent inter­actions in the crystal packing of (**I**). The Hirshfeld surface mapped over *d*_norm_ is represented in Fig. 4 ▸. The white surface indicates contacts with distances equal to the sum of van der Waals radii, and the red and blue colours indicate distances shorter or longer than the van der Waals radii, respectively. The bright-red spot near N1 indicates its role as a hydrogen-bond donor towards Cl1.

**Figure 5:**
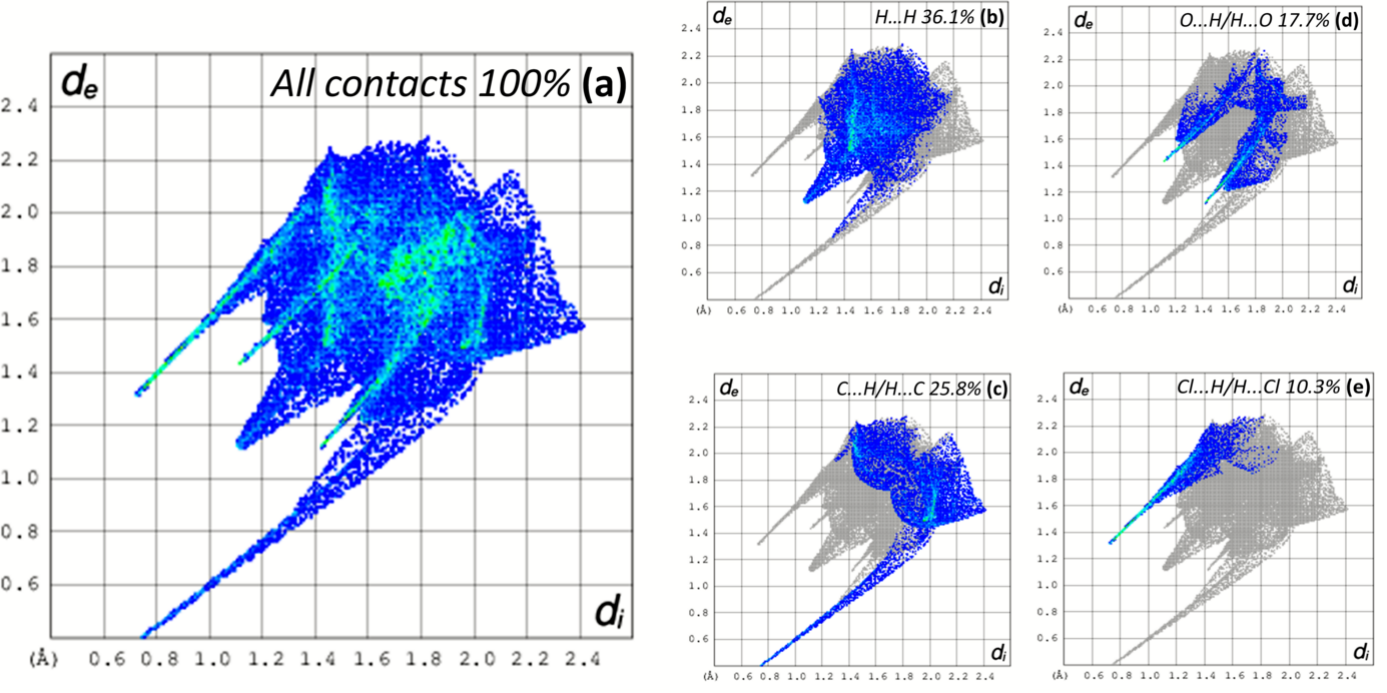
Two-dimensional fingerprint plots for the title compound, showing (*a*) all inter­actions, and decomposed into (*b*) H⋯H, (*c*) C⋯H/H⋯C, (*d*) O⋯H/H⋯O, (*e*) Cl⋯H/H⋯Cl inter­actions. Values for *d*_i_ and *d*_e_ represent the closest inter­nal and external distances (in Å) from given points on the Hirshfeld surface.

The most important contributions to the Hirshfeld surface arise from H⋯H contacts at 36.1% (Fig. 5 ▸*b*). C⋯H/H⋯C and O⋯H/H⋯O inter­actions follow with contributions of 25.8% and 17.7%, respectively (Fig. 5 ▸*c*,*d*). The classical N—H⋯Cl hydrogen bonds correspond to H⋯Cl/Cl⋯H contacts (10.3% contribution) and show up as a spike (Fig. 5 ▸*e*). Minor contributors are due to C⋯Cl/Cl⋯C (3.3%), N⋯H/H⋯N (2.4%), N⋯Cl/Cl⋯N (2.2%) and C⋯C (1.8%) inter­actions.

## 5. Database survey

A search of the Cambridge Structural Database (CSD, Version 5.43, last update November 2022; Groom *et al.*, 2016 ▸) for the 2-methyl­quinazolin-4(3H)-one moiety resulted in twelve hits with a similar planar conformation: ACANLC10 (Etter *et al.*, 1983 ▸), AWIYIR (Kalogirou *et al.*, 2021*a* ▸), BIHJUA and BIHKAH (Liao *et al.*, 2018 ▸), BOLGAK (Etter *et al.*, 1983 ▸) and BOYMAD (Chadwick & Easton, 1983 ▸), DILFEL (Rybarczyk-Pirek *et al.*, 2013 ▸), RUGTEV (Kalogirou *et al.*, 2020 ▸), UQOGAL (Kalogirou *et al.*, 2021*b* ▸) and YILLEM (Moghimi *et al.*, 2013 ▸). The main difference with respect to the mol­ecular structures of these compounds is that the C2—N1 bond in the pyrimidine ring of (**I**) is slightly longer due to the protonation of the N atom.

## 6. Synthesis and crystallization

30 g (0.2 mol) of *N*-acetyl­anthranilic acid and 76.53 g (1.4 mol) of ammonium chloride were placed in a 250 ml round-bottom flask. The mixture was heated in a sand bath at 498–503 K for 4 h. Then the reaction mixture was cooled and treated with boiling water. The mixture was filtered and brought to pH 7–9, and then was left at room temperature. The precipitate was filtered off, washed with distilled water and dried. Recrystallization from ethanol yielded 20.4 g (76%) of 2-methyl­quinazolin-4(3*H*)-one; m.p. 511–513 K, *R*_f_ = 0.28. In order to get 2-methyl­quinazolin-4(3*H*)-one hydro­chloride crystals, the latter was dissolved in a mixture of ethanol and methanol (9:1 *v*:*v*) to which 10 drops of 30%_wt_ HCl solution were added and stirred on a magnetic stirrer for 2 h. Crystal growth was carried out in a drying oven at 303 K. Colourless single crystals suitable for X-ray diffraction analysis were obtained after 5 d.

## 7. Refinement

Crystal data, data collection and structure refinement details are summarized in Table 2 ▸. H atoms were positioned geometrically (aromatic C—H = 0.93 Å, N—H = 0.86 Å and methyl C—H = 0.96 Å) and treated as riding atoms, with *U*_iso_(H) = 1.2*U*_eq_(aromatic C, N) or 1.5*U*_eq_(methyl C).

## Supplementary Material

Crystal structure: contains datablock(s) I. DOI: 10.1107/S2056989025000258/wm5743sup1.cif

Structure factors: contains datablock(s) I. DOI: 10.1107/S2056989025000258/wm5743Isup2.hkl

CCDC reference: 2416982

Additional supporting information:  crystallographic information; 3D view; checkCIF report

## Figures and Tables

**Table 1:** Hydrogen-bond geometry (Å, °)

*D*—H⋯*A*	*D*—H	H⋯*A*	*D*⋯*A*	*D*—H⋯*A*
N1—H1⋯Cl1	0.86	2.19	3.052 (2)	176
N3—H3⋯Cl1^i^	0.86	2.25	3.108 (3)	175

Symmetry code: (i) 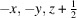.

**Table 2:** Experimental details

Crystal data	
Chemical formula	C_9_H_9_N_2_O^+^·Cl^−^
*M*_r_	196.64
Crystal system, space group	Orthorhombic, *Pbcm*
Temperature (K)	295
*a*, *b*, *c* (Å)	10.1221 (5), 13.6533 (4), 6.6248 (3)
*V* (Å^3^)	915.55 (7)
*Z*	4
Radiation type	Cu *K*α
μ (mm^−1^)	3.37
Crystal size (mm)	0.20 × 0.15 × 0.05

Data collection	
Diffractometer	PhotonJet (Cu) X-ray Source
Absorption correction	Multi-scan (*CrysAlis PRO*; Rigaku OD, 2020 ▸)
*T*_min_, *T*_max_	0.600, 1.000
No. of measured, independent and observed [*I* ≥ 2u(*I*)] reflections	7833, 977, 824
*R*_int_	0.086
(sin θ/λ)_max_ (Å^−1^)	0.616

Refinement	
*R*[*F*^2^ > 2σ(*F*^2^)], *wR*(*F*^2^), *S*	0.043, 0.133, 1.01
No. of reflections	977
No. of parameters	84
H-atom treatment	H atoms treated by a mixture of independent and constrained refinement
Δρ_max_, Δρ_min_ (e Å^−3^)	0.29, −0.35

Computer programs: *CrysAlis PRO* (Rigaku OD, 2020 ▸), *SHELXT* (Sheldrick, 2015 ▸), *OLEX2-refine* (Bourhis *et al.*, 2015 ▸) and *OLEX2* (Dolomanov *et al.*, 2009 ▸).

## supplementary crystallographic information

### 2-Methyl-4-oxo-3,4-dihydroquinazolin-1-ium chloride Crystal data

**Table:**

C_9_H_9_N_2_O^+^·Cl^−^	*D*_x_ = 1.427 Mg m^−^^3^
*M**_r_* = 196.64	Cu *K*α radiation, λ = 1.54184 Å
Orthorhombic, *P**b**c**m*	Cell parameters from 2575 reflections
*a* = 10.1221 (5) Å	θ = 4.4–70.9°
*b* = 13.6533 (4) Å	µ = 3.37 mm^−^^1^
*c* = 6.6248 (3) Å	*T* = 295 K
*V* = 915.55 (7) Å^3^	Prizm, colourless
*Z* = 4	0.20 × 0.15 × 0.05 mm
*F*(000) = 410.692	

### 2-Methyl-4-oxo-3,4-dihydroquinazolin-1-ium chloride Data collection

**Table:**

PhotonJet (Cu) X-ray Source diffractometer	824 reflections with *I*≥ 2u(*I*)
Detector resolution: 10.0000 pixels mm^-1^	*R*_int_ = 0.086
ω scans	θ_max_ = 71.7°, θ_min_ = 4.4°
Absorption correction: multi-scan (CrysAlisPro; Rigaku OD, 2020)	*h* = −12→12
*T*_min_ = 0.600, *T*_max_ = 1.000	*k* = −16→16
7833 measured reflections	*l* = −8→5
977 independent reflections	

### 2-Methyl-4-oxo-3,4-dihydroquinazolin-1-ium chloride Refinement

**Table:**

Refinement on *F*^2^	0 restraints
Least-squares matrix: full	14 constraints
*R*[*F*^2^ > 2σ(*F*^2^)] = 0.043	H atoms treated by a mixture of independent and constrained refinement
*wR*(*F*^2^) = 0.133	*w* = 1/[σ^2^(*F*_o_^2^) + (0.0787*P*)^2^ + 0.2674*P*] where *P* = (*F*_o_^2^ + 2*F*_c_^2^)/3
*S* = 1.01	(Δ/σ)_max_ = −0.0005
977 reflections	Δρ_max_ = 0.29 e Å^−^^3^
84 parameters	Δρ_min_ = −0.35 e Å^−^^3^

### 2-Methyl-4-oxo-3,4-dihydroquinazolin-1-ium chloride Fractional atomic coordinates and isotropic or equivalent isotropic displacement parameters (Å^2^)

**Table:**

	*x*	*y*	*z*	*U*_iso_*/*U*_eq_	
Cl1	0.15171 (8)	0.09775 (5)	0.75	0.0549 (3)	
O1	0.2374 (3)	0.60927 (16)	0.75	0.0819 (9)	
N1	0.1883 (2)	0.31959 (17)	0.75	0.0472 (6)	
H1	0.1741 (2)	0.25748 (17)	0.75	0.0708 (9)*	
C2	0.0870 (3)	0.3786 (2)	0.75	0.0463 (7)	
N3	0.1070 (3)	0.47530 (17)	0.75	0.0502 (6)	
H3	0.0384 (3)	0.51250 (17)	0.75	0.0753 (10)*	
C4	0.2314 (3)	0.5209 (2)	0.75	0.0558 (8)	
C5	0.4723 (3)	0.4864 (3)	0.75	0.0622 (9)	
H5	0.4897 (3)	0.5532 (3)	0.75	0.0746 (10)*	
C6	0.5757 (4)	0.4204 (3)	0.75	0.0679 (9)	
H6	0.6625 (4)	0.4428 (3)	0.75	0.0814 (11)*	
C7	0.5498 (4)	0.3204 (3)	0.75	0.0659 (9)	
H7	0.6197 (4)	0.2762 (3)	0.75	0.0791 (11)*	
C8	0.4220 (3)	0.2862 (2)	0.75	0.0584 (8)	
H8	0.4052 (3)	0.2192 (2)	0.75	0.0701 (10)*	
C9	0.3187 (3)	0.3528 (2)	0.75	0.0469 (7)	
C10	0.3423 (3)	0.4534 (2)	0.75	0.0491 (7)	
C11	−0.0492 (4)	0.3396 (3)	0.75	0.0610 (9)	
H11a	−0.058 (4)	0.274 (4)	0.75	0.0914 (13)*	
H11b	−0.090 (3)	0.360 (2)	0.869 (5)	0.0914 (13)*	

### 2-Methyl-4-oxo-3,4-dihydroquinazolin-1-ium chloride Atomic displacement parameters (Å^2^)

**Table:**

	*U*^11^	*U*^22^	*U*^33^	*U*^12^	*U*^13^	*U*^23^
Cl1	0.0623 (5)	0.0370 (4)	0.0655 (5)	−0.0071 (3)	−0.000000	0.000000
O1	0.0805 (18)	0.0339 (12)	0.131 (3)	−0.0025 (11)	−0.000000	0.000000
N1	0.0543 (14)	0.0323 (11)	0.0551 (14)	−0.0005 (10)	−0.000000	0.000000
C2	0.0531 (16)	0.0375 (13)	0.0483 (15)	0.0031 (12)	−0.000000	0.000000
N3	0.0558 (15)	0.0351 (12)	0.0597 (14)	0.0064 (11)	−0.000000	0.000000
C4	0.0652 (19)	0.0372 (15)	0.0651 (18)	−0.0022 (13)	−0.000000	0.000000
C5	0.065 (2)	0.0510 (18)	0.070 (2)	−0.0085 (16)	−0.000000	0.000000
C6	0.0526 (19)	0.071 (2)	0.080 (2)	−0.0055 (17)	−0.000000	0.000000
C7	0.0546 (19)	0.067 (2)	0.076 (2)	0.0093 (17)	−0.000000	0.000000
C8	0.0633 (19)	0.0425 (16)	0.069 (2)	0.0071 (14)	−0.000000	0.000000
C9	0.0555 (17)	0.0378 (14)	0.0474 (15)	0.0000 (13)	−0.000000	0.000000
C10	0.0566 (17)	0.0390 (14)	0.0518 (16)	−0.0038 (13)	−0.000000	0.000000
C11	0.0562 (19)	0.0480 (17)	0.079 (2)	0.0000 (15)	−0.000000	0.000000

### 2-Methyl-4-oxo-3,4-dihydroquinazolin-1-ium chloride Geometric parameters (Å, º)

**Table:**

O1—C4	1.208 (3)	C5—C10	1.391 (5)
N1—H1	0.8600	C6—H6	0.9300
N1—C2	1.304 (4)	C6—C7	1.389 (5)
N1—C9	1.396 (4)	C7—H7	0.9300
C2—N3	1.335 (4)	C7—C8	1.375 (5)
C2—C11	1.478 (5)	C8—H8	0.9300
N3—H3	0.8600	C8—C9	1.386 (4)
N3—C4	1.404 (4)	C9—C10	1.394 (4)
C4—C10	1.452 (5)	C11—H11a	0.91 (5)
C5—H5	0.9300	C11—H11b^i^	0.93 (3)
C5—C6	1.381 (5)	C11—H11b	0.93 (3)
			
C2—N1—H1	118.56 (17)	H7—C7—C6	119.6 (2)
C9—N1—H1	118.56 (15)	C8—C7—C6	120.8 (3)
C9—N1—C2	122.9 (2)	C8—C7—H7	119.6 (2)
N3—C2—N1	119.5 (3)	H8—C8—C7	120.4 (2)
C11—C2—N1	120.7 (3)	C9—C8—C7	119.1 (3)
C11—C2—N3	119.9 (3)	C9—C8—H8	120.43 (19)
H3—N3—C2	117.49 (17)	C8—C9—N1	120.1 (3)
C4—N3—C2	125.0 (3)	C10—C9—N1	118.8 (3)
C4—N3—H3	117.49 (16)	C10—C9—C8	121.1 (3)
N3—C4—O1	119.2 (3)	C5—C10—C4	121.7 (3)
C10—C4—O1	126.5 (3)	C9—C10—C4	119.5 (3)
C10—C4—N3	114.3 (2)	C9—C10—C5	118.7 (3)
C6—C5—H5	119.8 (2)	H11a—C11—C2	117 (3)
C10—C5—H5	119.8 (2)	H11b—C11—C2	107.9 (19)
C10—C5—C6	120.4 (3)	H11b^i^—C11—C2	107.9 (19)
H6—C6—C5	120.1 (2)	H11b^i^—C11—H11a	105 (2)
C7—C6—C5	119.8 (3)	H11b—C11—H11a	105 (2)
C7—C6—H6	120.1 (2)	H11b—C11—H11b^i^	116 (4)
			
O1—C4—N3—C2	180.0	N3—C4—C10—C5	180.0
O1—C4—C10—C5	0.0	N3—C4—C10—C9	0.0
O1—C4—C10—C9	180.0	C4—C10—C5—C6	180.0
N1—C2—N3—C4	0.0	C4—C10—C9—C8	180.0
N1—C9—C8—C7	180.0	C5—C6—C7—C8	0.0
N1—C9—C10—C4	0.0	C5—C10—C9—C8	0.0
N1—C9—C10—C5	180.0	C6—C7—C8—C9	0.0
C2—N3—C4—C10	0.0	C7—C8—C9—C10	0.0

Symmetry code: (i) *x*, *y*, −*z*+3/2.

### 2-Methyl-4-oxo-3,4-dihydroquinazolin-1-ium chloride Hydrogen-bond geometry (Å, º)

**Table:**

*D*—H···*A*	*D*—H	H···*A*	*D*···*A*	*D*—H···*A*
N1—H1···Cl1	0.86	2.19	3.052 (2)	176
N3—H3···Cl1^ii^	0.86	2.25	3.108 (3)	175

Symmetry code: (ii) −*x*, −*y*, *z*+1/2.
